# A comparative study on the dose–effect of low-dose radiation based on microdosimetric analysis and single-cell sequencing technology

**DOI:** 10.1038/s41598-024-62501-5

**Published:** 2024-05-21

**Authors:** Yidi Wang, Jin Gao, Bo Tang, Wei Mo, Han Gao, Jiahao Guo, Xianghui Kong, Wenyue Zhang, Yuchen Yin, Yang Jiao, Liang Sun

**Affiliations:** 1 State Key Laboratory of Radiation Medicine and Protection, School of Radiation Medicine and Protection, Soochow University, Suzhou, 215123 China; 2 Department of Public Health Surveillance and Evaluation, Shandong Center for Disease Control and Prevention, Jinan, 250014 China

**Keywords:** Low dose radiation, Microdosimetry, Monte Carlo, Mesh-type cell model, Single-cell sequencing, Biological physics, Nuclear physics, Molecular biophysics, Computational biology and bioinformatics

## Abstract

The biological mechanisms triggered by low-dose exposure still need to be explored in depth. In this study, the potential mechanisms of low-dose radiation when irradiating the BEAS-2B cell lines with a Cs-137 gamma-ray source were investigated through simulations and experiments. Monolayer cell population models were constructed for simulating and analyzing distributions of nucleus-specific energy within cell populations combined with the Monte Carlo method and microdosimetric analysis. Furthermore, the 10 × Genomics single-cell sequencing technology was employed to capture the heterogeneity of individual cell responses to low-dose radiation in the same irradiated sample. The numerical uncertainties can be found both in the specific energy distribution in microdosimetry and in differential gene expressions in radiation cytogenetics. Subsequently, the distribution of nucleus-specific energy was compared with the distribution of differential gene expressions to guide the selection of differential genes bioinformatics analysis. Dose inhomogeneity is pronounced at low doses, where an increase in dose corresponds to a decrease in the dispersion of cellular-specific energy distribution. Multiple screening of differential genes by microdosimetric features and statistical analysis indicate a number of potential pathways induced by low-dose exposure. It also provides a novel perspective on the selection of sensitive biomarkers that respond to low-dose radiation.

## Introduction

Low-dose ionizing radiation typically does not lead to rapid cell death or immediate damaging response in tissues (organs). However, it may increase an individual's long-term risk for carcinogenesis and genomic instabilities^1^ and is associated with non-cancer health outcomes such as cardiovascular diseases, neurological disorders, immune function disorders, and cataracts^2^. The linear no-threshold (LNT) model is currently the standard model used to describe the relationship between radiation dose and cancer risk^3^. Its prediction results are consistent with epidemiological data from atomic bomb survivors in Japan^4–6^. Additionally, in vitro studies have identified bystander effects and adaptive responses, suggesting a nonlinear induction of low-dose radiation effects^7–10^. Nevertheless, experimental results and epidemiological surveys indicated a nonlinear relationship between low-dose biological effects and radiation dose^11–13^, which contradicts the assumptions of the LNT model.

Under low-dose irradiation conditions (defined as doses below 100 mGy or low dose rates below 5 mGy/h)^1^, common cellular damage (e.g., cell survival fractions or DNA damage) cannot directly be observed by conventional experimental methods compared to high-dose radiation. Instead, phenomena like bystander effects, radiation hormesis, and adaptive responses to low-dose radiation have been observed^14–21^. Given the diversity of low-dose biological effects, identifying them in a mutually supportive manner is challenging.

Due to the stochastic nature of radiation interactions with matter, there are notable variations in the distribution of radiation energy deposition at the microscopic level^22^. Many biophysical models have established links between radiation dose and biological effects, considering the stochastic nature of radiation effects^23–26^. For low-dose radiation, there is a plausible assumption that the numerical uncertainty in microscopic doses significantly contributes to the uncertainty in biological effects^27,28^. Therefore, it is necessary to explore methods and indicators that can characterize this uncertainty in terms of radiation dosimetry and biological effects.

From the perspective of physical dosimetry, macroscopic dose indicators can only reflect the overall average dose in the irradiated material. As a result, capturing statistical fluctuations within microscopic volumes is challenging, especially in the case of low-dose radiation when influenced by the stochastic nature of interactions. Furthermore, microdosimetry theory can characterize the statistical fluctuations in the microscopic volume of radiation energy deposition by quantifying dose distributions at the micrometer scale^29^. Some studies have already demonstrated the uncertainty of cell-scale dose distribution. However, these studies typically used simple geometric shapes (spheres, ellipsoids, cubes, etc.) to represent cell shapes^25,27,30–32^. This approach may affect the accuracy of cellular dose estimation to some extent due to the irregular morphology of realistic cells. By combining the previously established cell mesh-type models and Monte Carlo (MC) methods in our previous work, the "actual dose" (specific energy) within the individual cell nucleus and their dose distribution among the cell population resulting from low-dose exposure can be determined^33,34^.

In terms of biological effects, traditional methods used in radiation biology experiments, such as survival fraction and DNA double-strand or single-strand break yields, can only provide a general assessment of the damage suffered by all cells within a given sample. Under the same irradiation conditions, these methods cannot capture the heterogeneity in the response of irradiated individual cells within samples, especially for low-dose radiation. The 10 × Genomics single-cell sequencing technology enables precise mapping of the biological information triggered by low-dose radiation to each cell, facilitating accurate identification of changes in gene expression levels within cell populations induced by low-dose radiation^35,36^. Therefore, single-cell sequencing technology provides insights into the gene expression levels of individual cells exposed to low doses, thus revealing the distribution of differential gene expressions within these cell populations, which represents uncertainties of biological responses.

This study improves the cell mesh-type models from our previous works^33,34^. The factors influencing the distribution of cellular doses resulting from external exposure to photon beams were evaluated through a monolayer mesh-type cell population model and the MC method. Through single-cell sequencing, the cell line exposed to low-dose radiation was examined to reveal variations in gene expression among different irradiation groups. The characteristics of nucleus dose distribution obtained from microdosimetric analysis were used as indicators to identify differential genes, which were then subjected to essential bioinformatics analysis. Moreover, this study attempted to establish an effective connection between the "nucleus-specific energy distribution" and the "differential gene expression distribution". This connection can facilitate the exploration and development of biomarkers that are highly sensitive to low-dose radiation, thereby revealing the mechanisms of the molecular biological effects under low-dose radiation conditions using microdosimetric methods.

## Materials and methods

Lung injury induced by radiation is a common side effect of radiotherapy. Hence, the human normal lung epithelial cell line (BEAS-2B) was selected as the research subject in this study. The cell-specific energy distribution and gene expression distribution within a cell population induced by low linear energy transfer (LET) photon external irradiation were investigated through simulations and experiments. With the development of the cell computational model, MC simulations, and microdosimetric analysis, the cell-specific energy distribution was acquired. Furthermore, the gene expression distribution was obtained by performing single-cell sequencing on irradiated cells. Finally, the results were compared to identify differential genes that met both microdosimetric distribution characteristics and statistical significance criteria, which were then subjected to bioinformatics analysis.

### Constructions of monolayer cell population models in PHITS

**Figure 1:**
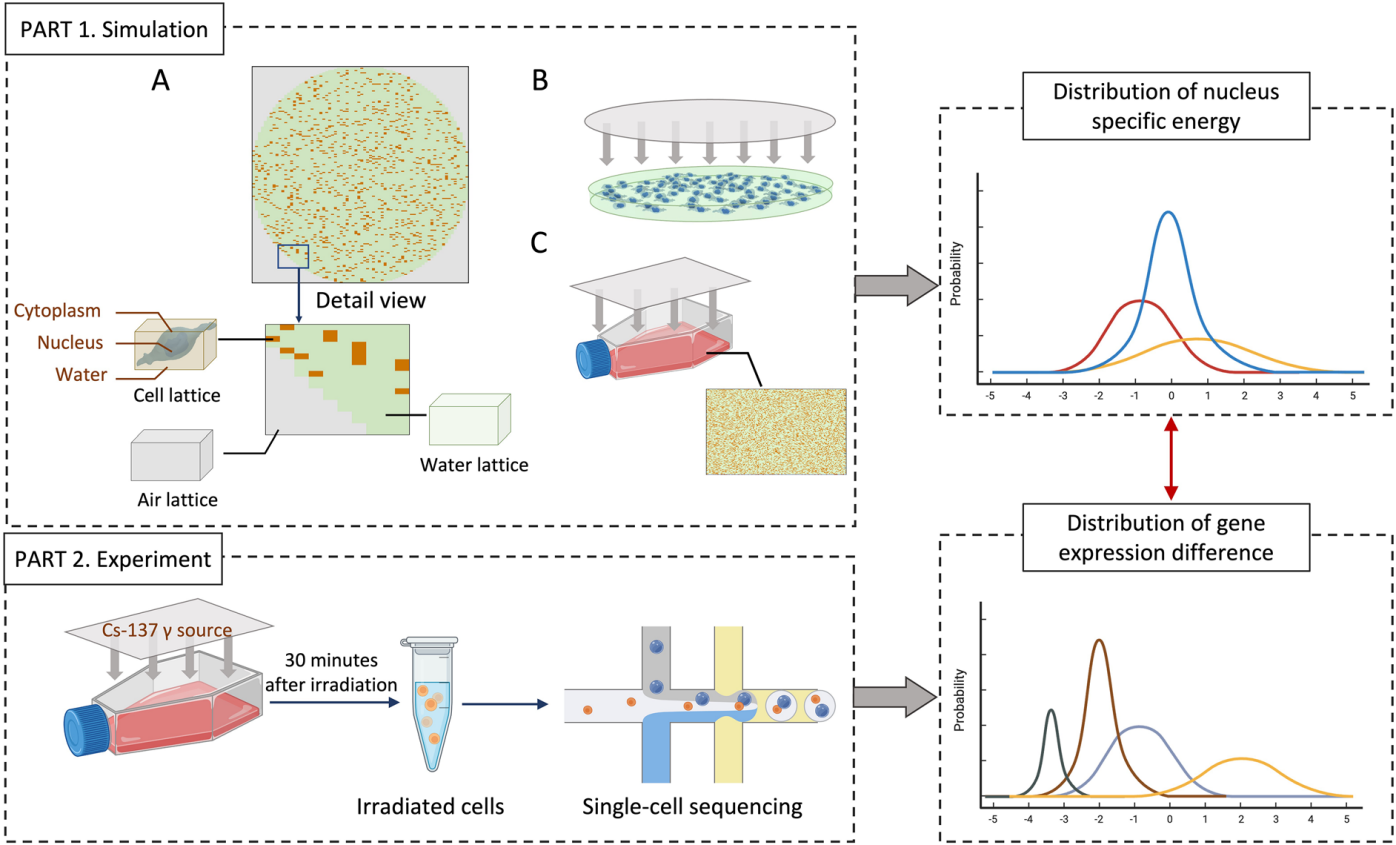
The schematic diagram of the simulation and experimental workflow. Part 1 outlines the process of constructing a monolayer cell population model. (**A**) is an overhead view of the monolayer cell population model along with an enlarged detail. (**B**) and (**C**) represent schematic illustrations of Monte Carlo simulations for irradiation scenarios in circular and square culture dishes containing the monolayer cell population model (the gray planes indicate the monoenergetic photon external irradiation source). Part 2 illustrates the workflow for cell irradiation and single-cell sequencing. A connection is established between these two categories of distributions obtained through Part 1 and Part 2. The Monte Carlo simulations from Part 1 provide the specific energy distribution within cell nucleus, while the sequencing results from Part 2 reveal the distribution of gene expression differences.

The Particle and Heavy Ion Transport code System (PHITS) is a general-purpose MC radiation transport code that can simulate the behavior of most particle species, which allows for definitions of tetrahedron geometry^37^. Since the cellular dose is significantly influenced by shape and volume, it is inadequate to rely solely on regular geometries such as spheres or cubes when assessing cellular dose^32,34^. The mesh-type cell model is a kind of tetrahedral model that can be directly imported into PHITS for estimating cellular doses, which is reconstructed from laser confocal tomography images of stained cells. Based on our preliminary work, we constructed a monolayer mesh-type cell population model that approximates the replicates of in vitro cell culture scenarios. The monolayer mesh-type cell population model comprised three distinct "lattices" including the "Cell lattice", the "Water lattice", and the "Air lattice", representing the positions and materials of cells inside the petri dish, the culture medium inside the dish, and the air surrounding the round-bottomed petri dish, respectively. It's worth noting that the size of "lattices" should be slightly larger than the overall volume of the cells. Depending on different objectives, the "lattices" can be combined to monolayer cell population models with different shapes and number densities. In this work, circular monolayer cell population models (Fig. 1, PART 1-panel B) and square monolayer cell population models (Fig. 1, PART 1-panel C) were constructed. The diameter of the circular cell population model was approximately 6.4 mm (corresponding to a 96-well plate well), which holds a total of 1134 cells. In the square cell population models, two sizes (approximately 5.9 mm × 3.9 mm and 1.2 mm × 0.8 mm) and two number densities (8000 cells and 1500 cells) were considered.

In order to assess the sensitivity of cellular morphological parameters (e.g., volume and shape) to the dose distribution within cell populations, similar mesh-type models and simplified geometric models were constructed for comparison. The simplified geometric model is referred to as the "Geometry-type model", which replaces the mesh-type model in the "Cell lattice" of Fig. 1, PART1-panel A with concentric ellipsoids. The projected dimensions of these concentric ellipsoids in the three-dimensional coordinate system are the same as for the mesh-type model, and the dimensional parameters of the cell models are listed in the Supplementary Material S.1.

### Monte Carlo simulations

This study used PHITS version 3.30 for MC simulations. The mesh-type model was in a tetrahedral geometry format and was imported through TetGen format files with ".ele" and ".node" extensions. The EGS model was employed to simulate and record the transport of radiation particles^38^. To ensure sampling efficiency, the radiation source was set to slightly larger than the dimensions of the monolayer cell population model, as shown in panels B and C of Fig. 1, PART 1. For the circular cell population model (as shown in Fig. 1, PART1-panel B), the source was initialized with monoenergetic photons of energies 0.1, 0.2, 0.5, and 1 MeV. For the square cell population model (as shown in Fig. 1, PART1-panel C), the source was set to Cs-137 external irradiation photons with an energy of 661.7 keV. The cutoff energies for photons and electrons were set as 1 keV. Afterward, the dose distributions within the petri dish for different cumulative absorbed doses on cells and their nucleus were calculated. The number of particles in each simulation varies depending on cumulative doses, as detailed in Supplementary Material S3. In order to obtain specific energies at different expected cumulative macroscopic dose levels, it is necessary to multiply the direct output of the [t-deposit] tally of the energy deposition or dose results renormalized by particle number by the corresponding particle number. The dose per cell nucleus or cytoplasm within a cell population and its systematic error can be directly obtained from the PHITS results. The specific energies of individual cells are then divided into several bins from low to high to form a frequency distribution. However, the errors associated with frequency distributions cannot be directly obtained by PHITS. Therefore, we performed three separate runs for each simulation to statistically determine the frequencies corresponding to each energy bin. The error bars represent the standard deviation of the probabilities for each bin of the distribution obtained from the three calculations.

In the monolayer cell population model, the "water lattice" was made of liquid water (H_2_O, 1 g/cm^3^), The material surrounded by the round-bottomed petri dish ("Air lattice") was set as air. The material of the cell nucleus and cytoplasm in the "Cell lattice" is based on the work of Incerti et al., as detailed in Supplementary Material S2^39^.

### Microdosimetric assessment

In microdosimetry theory, the concept of specific energy (denoted as $z$) is used to express the actual dose deposited per unit mass in a given microvolume, i.e., $z=\varepsilon /m$, where $\varepsilon$ represents the imparted energy (in J), and $m$ is the mass of the microvolume (in kg). The specific energy distribution within a site caused by a single event is represented as ${f}_{1}\left(z\right)$, which encompasses all the interaction processes initiated by an initial particle and its secondary electrons. To achieve a certain cumulative dose at a macroscopic level, it is necessary to consider specific energy distributions resulting from numerous initial particles (i.e., multiple events). When the absorbed dose is denoted as $D$, the frequency distribution function of specific energy $z$ can be expressed by:

1 $$F\left(z,D\right)=P(Z\le z,D)$$

The differential form $f\left(z,D\right)$ can be expressed by:

2 $$f\left(z,D\right)=dF\left(z,D\right)/dz$$

where $f\left(z,D\right)$ represents the probability of having a specific value of specific energy $z$ for an absorbed dose of $D$; $f\left(z,D\right)*dz$ represents the probability of specific energy falling within the range of $z$ to $z+dz$. In this study, the cell nucleus was considered as the sensitive volume for quantifying the energy deposition characteristics within the cell population. On this basis, the specific energy distribution for the cell nucleus and its corresponding mean value can be obtained, denoted as $f\left(z,D\right)$ and $\overline{z }$, respectively. To assess the statistical variability of the distribution, the ratio of the standard deviation to the mean value of $f\left(z,D\right)$ was employed, represented as $\sigma /\overline{z }$, which is referred to as the statistical variability of the distribution. $\sigma$ represents the standard deviation of specific energies of each cell nucleus or cytoplasm within the cell population.

By convolving the energy spectrum of a single event, the energy spectrum of multiple events can be obtained:

3 $${f}_{v}\left(z\right)={\int }_{0}^{{z}_{max}}{f}_{v-1}(z-{z}^{\prime}){f}_{1}({z}^{\prime})d{z}^{\prime}={f}_{1}\left(z\right)*{f}_{v-1}\left(z\right)$$

where ${f}_{v}\left(z\right)$ represents the specific energy frequency distribution obtained by convolving the specific energy frequency distribution of a single event ${f}_{1}\left(z\right)$ by $v$ times. The value of $v$ depends on the desired macroscopic dose level. Assuming that the total dose level caused by a single initial particle is ${D}_{1}$, then when the cumulative dose reaches $D$, $v$ can be determined as follows

4 $$v=D/{D}_{1}$$

Note that $v$ should be an integer. ${D}_{1}$ also refers to the convolved distributions with a certain dose value. Detailed explanations of convolution integrals can be found in Supplementary Material S4.

$f\left(z,D\right)$ represents a distribution that reflects fluctuations in the energy deposition by radiation particles within a microscopic volume. The specific energy distribution of cells is a continuous probability distribution function (PDF), which tends to exhibit a shape similar to that of a normal distribution. To quantitatively compare the differences in specific energy distributions under different conditions with a normal distribution, normal distributions were generated that can be used for comparison with the specific energy distributions. The mean and standard deviation of the normal distribution are derived from the specific energy distribution obtained from Monte Carlo simulations.

To quantify the differences between specific energy distributions by PHITS, convolution distributions, and normal distribution, the root mean square error (RMSE) is calculated:

5 $$RMSE=\sqrt{\left[\sum_{i=1}^{N}{\left({f}_{i,reference}-{f}_{i,MC}\right)}^{2}\right]/N}$$

where ${f}_{i,MC}$ represents the specific energy frequency distribution $f\left(z,D\right)$ obtained from MC simulations, $N$ is the number of bins in $f\left(z,D\right)$, and ${f}_{i,reference}$ represents convolution or normal distributions. RMSE was used as a metric to ensure comparability and facilitate comparisons under the same parameters. In order to achieve meaningful horizontal comparisons, it is necessary to ensure that the bin lengths remain uniform.

### Cell irradiation conditions and single-cell sequencing

In this study, the BEAS-2B cell line was exposed to the Cs-137 gamma-ray source. The Cs-137 irradiation equipment was designed and manufactured by Hopewell Designs company. The irradiation doses were 10 mGy, 100 mGy, and 1 Gy, where 10 mGy and 100 mGy belonged to the low-dose group, while 1 Gy belonged to the high-dose group. The dose rate was maintained at 100 mGy/min, and the irradiation was conducted using the T25 culture bottle.

Irradiated cells cultured for 30 min were processed by 10 × Genomics single-cell sequencing, as illustrated in Fig. 1, Part 2. This process produced a two-dimensional matrix comprising cells and their corresponding gene expression levels. A total of 23,517 genes were collected for each group, each containing approximately 10,000 cells.

### Multiple screening of differential genes and bioinformatics analysis

Frequency distributions of gene expression differences were separately generated for the low-dose group (10 mGy and 100 mGy). In addition, these expression differences were calculated by dividing the gene expression values of each cell by the mean expression of that gene in the control group.

The selection of differential genes was performed in three steps. The first step was to statistically compare the mean gene expression values between irradiated and control groups to identify differentially expressed genes. Differential gene selection was performed by calculating the mean expression for each gene and applying filtering criteria based on fold change (FC) and statistical significance (p-value). The thresholds for FC and p-values were set at 1.2 and 0.05, respectively. In order to further analyze the data, we used RStudio version 3.6.2 and the ClusterGVis package. The transcriptome data from each series was clustered into distinct clusters using the mfuzz algorithm. Heatmaps and box plots were generated for each cluster to visualize trends and identify differentially expressed genes related to the dose. Additionally, Cytoscape version 3.9.1 and the MCODE plugin were used to select protein–protein interaction (PPI) network modules^40^. The criteria for selecting modules include a degree cutoff of 2, a node score cutoff of 0.2, a k-core of 2, and a maximum depth of 100. Finally, the CytoHubba plugin^41^ was used in the target network to calculate the node scores in a computational mode to select the top 100 hub genes, referred to as "Cytoscape merge".

The second step focused on selecting differential genes that exhibit features consistent with microdosimetric distributions. Simulation results of cellular-specific energy distributions revealed that the dispersion of specific energy distributions decreases with increasing cumulative dose. Based on this pattern, it could be concluded that the criterion for screening differential genes (referred to as "microdosimetric merge") was that their expression differences show decreased dispersion with increasing irradiation dose. Genes meeting both criteria were further ranked based on their RMSE values between gene expression distribution and their normal distribution with the same mean value and standard deviation. Finally, the top 50 genes (termed as "multi-filtered" genes) were selected for subsequent bioinformatics analysis.

Subsequently, differential gene enrichment analysis was performed using RStudio version 3.6.2. The analysis involved generating the top 10 team plots for GO and KEGG analysis using the GOplot R and ggplot software packages, respectively^42–45^.

## Results

### Characteristics of nucleus-specific energy distribution

**Figure 2:**
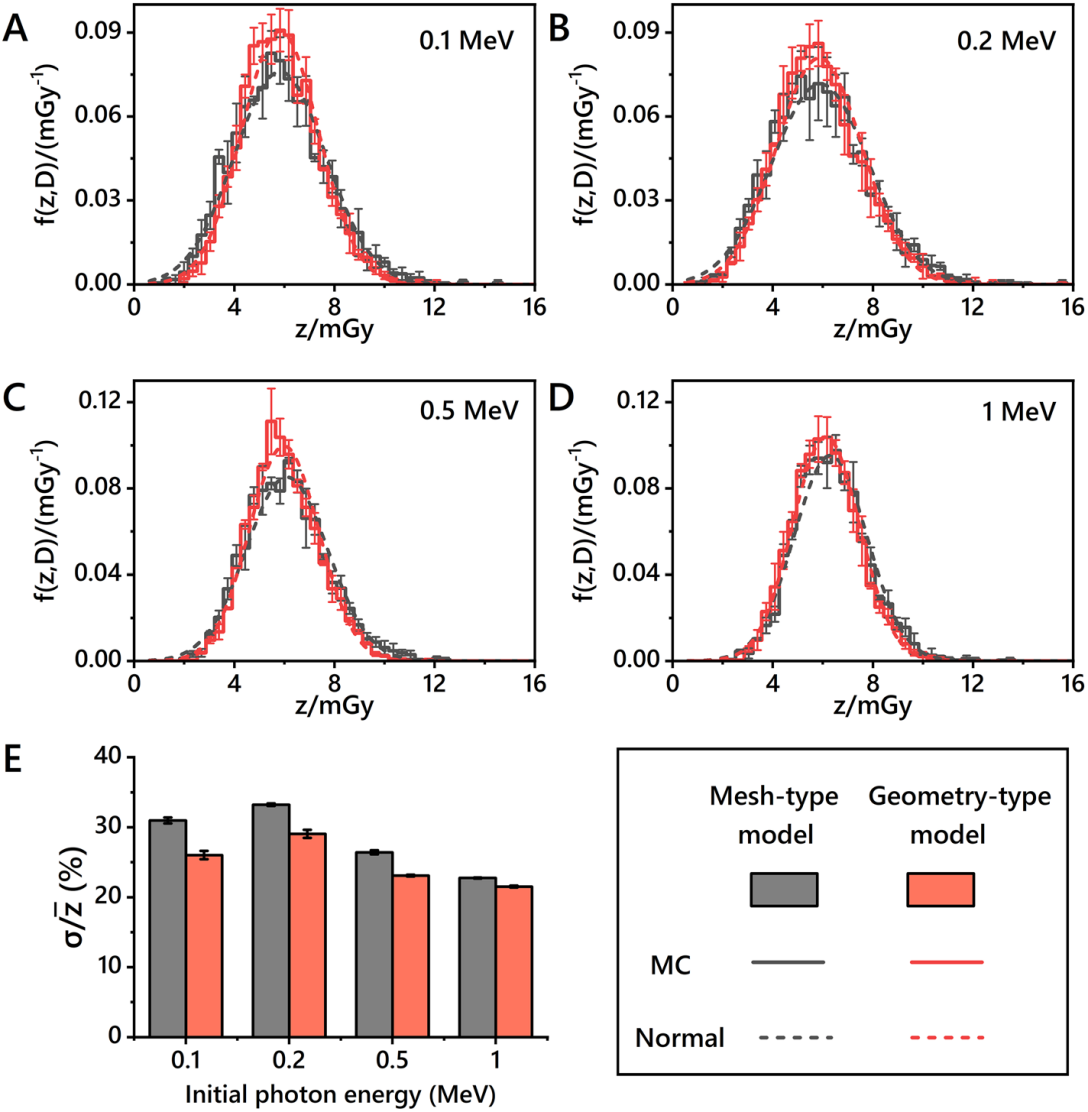
Specific energy distributions of cell nucleus in the monolayer cell population model for monoenergic photons with incident energies of 0.1 MeV (**A**), 0.2 MeV (**B**), 0.5 MeV (**C**), and 1 MeV (**D**) at the macroscopic dose level of 5 mGy. The mesh-type (black) and geometric-type (red) models are considered. The solid lines represent the actual specific energy distributions, while the dashed lines represent the normal distributions by the mean and standard deviation of the specific energy distribution obtained from MC simulations. Panel E provides a comparison of the dispersion of specific energy distributions obtained from two types of cell population models.

Figure 2 illustrates the specific energy distribution of the cell nucleus (solid lines) in a monolayer cell population model caused by monoenergetic photons with 4 types of incident energies (0.1 MeV, 0.2 MeV, 0.5 MeV, and 1 MeV) at the same macroscopic dose level (5 mGy). It also compares the distribution differences caused by different types of cell models (i.e., the geometric-type model and the mesh-type model). The Supplementary Material S5 displays the mean and standard deviation of specific energy distributions for different parameters. The normal distributions (dashed lines) are plotted based on the same mean and standard deviation for each MC distribution. RMSE values calculated by comparing various distributions are listed in the supplementary materials S8. RMSE values between MC results and normal distributions exhibit minimal sensitivity to the incident energy, mostly around 4 × 10^–3^. The average specific energy of the cell nucleus within the cell population is not equivalent to the macroscopic dose level. In this context, the macroscopic dose level represents the cumulative average absorbed dose within the culture medium of the petri dish with liquid water and without cells. Regardless of the type of cell model, the average specific energy of the cytoplasm or cell nucleus is generally 1.2 times the macroscopic average dose, and this ratio slightly increases with increasing photon incident energies. However, there is no direct correlation between dispersion and incident energy. There are also slight differences between the macroscopic dose with different materials as shown in the supplementary material S3. The macroscopic doses of petri dish filled with geometry-type or mesh-type cells with the cell materials are different from those with liquid water. The type of cell model within a cell population can influence the macroscopic dose of culture dish, but it does not affect the average specific energy of the cell nucleus or cytoplasm.

Compared with the geometric-type model, the mesh-type model exhibits higher dispersion in the specific energy distribution of both the cytoplasm and cell nucleus. With photons of an initial energy of 0.1 MeV, the dispersions of specific energy distributions for the cytoplasm and nucleus in the mesh-type cell population model are 15.08% and 30.98%, respectively, while those in the geometric-type model are 11.66% and 26.02%, respectively. More detailed information regarding the differences in the distribution of the cell nucleus and cytoplasm can be found in Supplementary Material S5. Combining the distributions from Fig. 2 and S6 and the dispersion values listed in S5, it can be seen that the model type affects the dispersion of the cell nucleus or cytoplasm-specific energy distribution.

**Figure 3:**
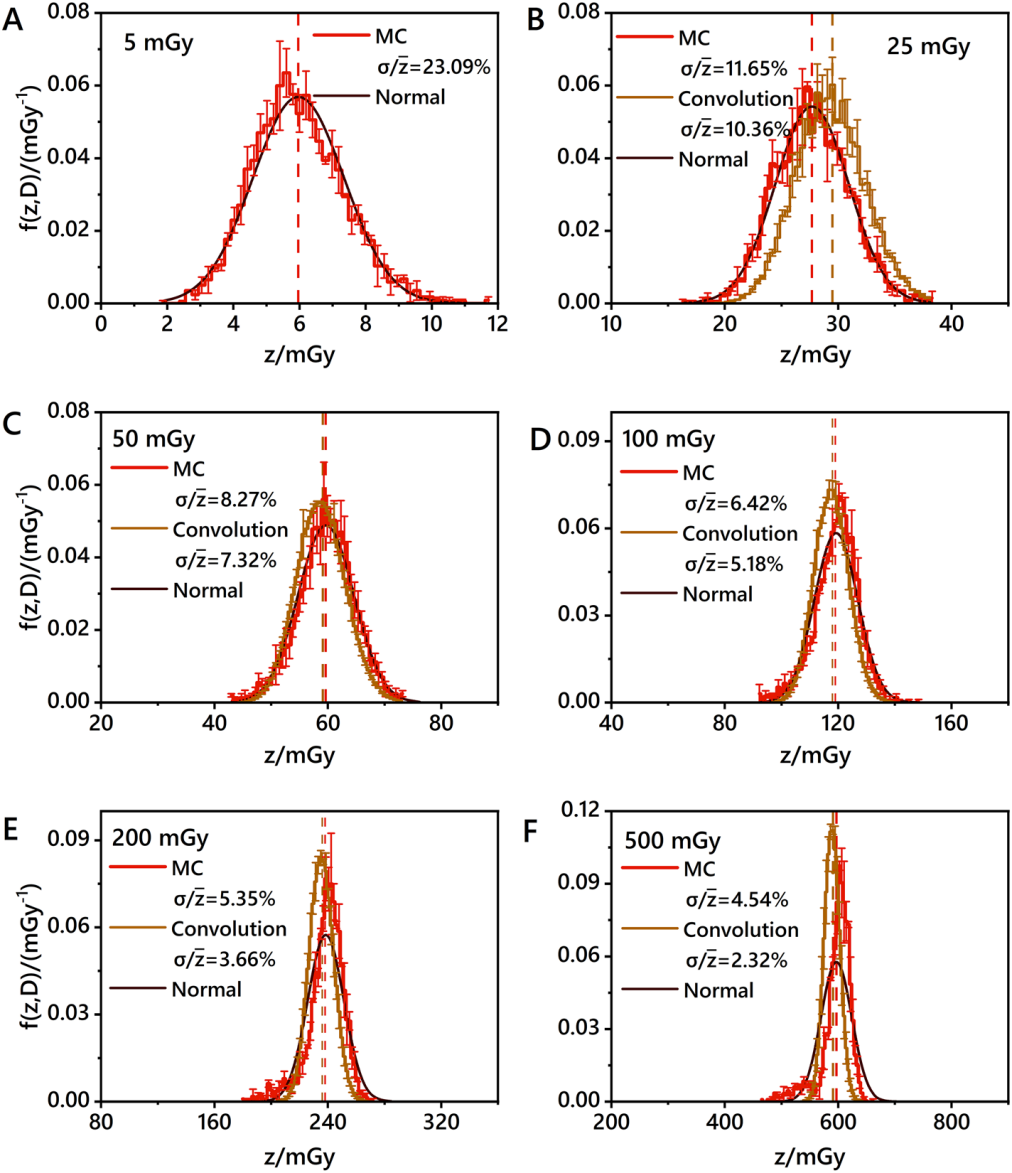
Specific energy distributions of cell nucleus in a monolayer geometric-type cell population model for monoenergetic photons with an incident energy of 0.5 MeV at cumulative macroscopic dose levels of 5 mGy (**A**), 25 mGy (**B**), 50 mGy (**C**), 100 mGy (**D**), 200 mGy (**E**), and 500 mGy (**F**). The red line represents the specific energy distribution obtained by MC simulations. Brown lines represent specific energy distributions obtained through convolution with different times, starting from the specific energy distribution of cell nucleus at a cumulative dose of 5 mGy, which was obtained through MC simulation. Black lines represent normal distributions by the mean and standard deviation of the specific energy distribution obtained from MC simulations.

Figure 3 presents the cell nucleus-specific energy distribution within a monolayer geometric-type cell population model at 6 different cumulative macroscopic dose levels, ranging from 5 to 500 mGy. These distributions are accompanied by normal distributions with the same mean and standard deviation as specific energy distributions. The specific energy distribution at a cumulative dose of 5 mGy obtained through MC simulation (Fig. 3A) is the convolved distribution, which means other convolution distributions in Fig. 3B–F are derived from the MC distribution in Fig. 3A by convolution integral. With different convolution times, nucleus-specific energy distributions at varying cumulative dose levels were obtained. The dispersion of the specific energy distribution decreased with increasing cumulative dose, indicating that the cell nucleus-specific energy had more statistical fluctuations at lower dose levels. The average specific energy of the cell nucleus, whether obtained through MC simulation or convolution, remained constant at any macroscopic cumulative doses.

Distributions generated by convolution generally reflect the characteristics of specific energy distribution obtained from MC simulations. However, there are some differences between these two distributions. In terms of dispersion, the deviation increases as the number of convolutions increases. This deviation is primarily characterized by an underestimation of dispersion in the convolution distribution. At a cumulative dose of 500 mGy, the dispersion of the specific energy distribution obtained through MC simulation is 4.54%, while that of the convolution distribution's dispersion is 2.32%. The RMSE values also reflect similar trends. As shown in S8.3, the RMSE between MC and convolution increases with increasing cumulative dose. However, the convolution algorithm cannot predict specific energy distribution at a cumulative dose of 25 mGy because the mean value is significantly biased (Fig. 3). Moreover, RMSE values between normal and convolution distributions are greater than those between MC and convolution distributions. Convolution distributions appear to be more symmetric around the mean value.

As can be seen from S8.3, under the same geometric conditions, the RMSE value between MC and normal distributions does not reach its maximum at a cumulative dose of 1 mGy. However, it exceeds the values observed at low doses (cumulative dose of 5 mGy) for other conditions, as indicated in S8.1 and S8.2. This difference also leads to the noticeable differences for very low macroscopic dose (1 mGy) between MC and normal distributions in S7 (Panels A, C, F). As specific energies cannot be negative, even if the specific energy distribution is similar to the normal distribution at a macroscopic dose of 1 mGy (symmetrical around a certain value), this value is not equal to the mean specific energy. Conversely, the normal distribution with the same mean and standard deviation only encompasses the portion of its abscissa greater than 0.

At high dose levels, MC simulation results tend to exhibit higher frequencies within narrower specific energy ranges. In contrast, the distribution obtained through convolution is less similar to the normal distribution according to S8.2. To assess the impact of petri dish geometry factors such as shape, size, and cell number density on the specific energy distribution of the cell nucleus, the specific energy distribution of the cell nucleus under Cs-137 external photon irradiation at various cumulative doses was obtained, as shown in Supplementary Material S7. The results indicate that the dispersion of the specific energy distribution of the cell nucleus within a monolayer cell population is unaffected by geometric factors under uniform radiation from the same source. In a large square petri dish (5.9 mm × 3.9 mm) with 8000 cells and a cumulative dose of 10 mGy, the dispersion of the distribution is 15.55%, with a mean specific energy of 12.13 mGy. For petri dishes with 8000 and 1500 cells, the corresponding values are 15.30%, 11.46 mGy, and 15.16%, 10.95 mGy, respectively. Additionally, the observed trend remains consistent among petri dishes of different geometries: the dispersion decreases as the cumulative dose increases. The convolution method can also be used to predict the specific energy distribution at high cumulative doses based on the distribution at lower cumulative doses. For instance, the dispersion of the specific energy distribution obtained through MC simulation is 5.02% at a cumulative dose of 1 mGy, and the convolution-derived distribution is 4.86% at a cumulative dose of 100 mGy.

### Differential gene expression distributions in the low-dose group

Based on the results of MC simulation and microdosimetric analysis, under low cumulative dose conditions, the nucleus-specific energy distribution potentially exhibits a pattern approximating a normal distribution, and its dispersion decreases with the increasing dose. In light of this insight, we statistically analyzed the dispersion of gene expression differences in the low-dose group and selected the genes demonstrating "expression difference distribution dispersion decreasing with increasing cumulative dose". Subsequently, the mean and standard deviations of the expression distribution were deduced to a normal distribution, and the RMSE was calculated between the expression difference dispersion distribution and the normal distribution. A smaller RMSE indicates a closer approximation to a normal distribution. The selected genes were categorized into the low-dose group (10 mGy and 100 mGy) based on the compared RMSE between expression difference distribution and normal distribution, and the top 100 differentially expressed genes were chosen. For more details, please refer to the "Microdosimetric merge" in Supplementary Material S9.

**Figure 4:**
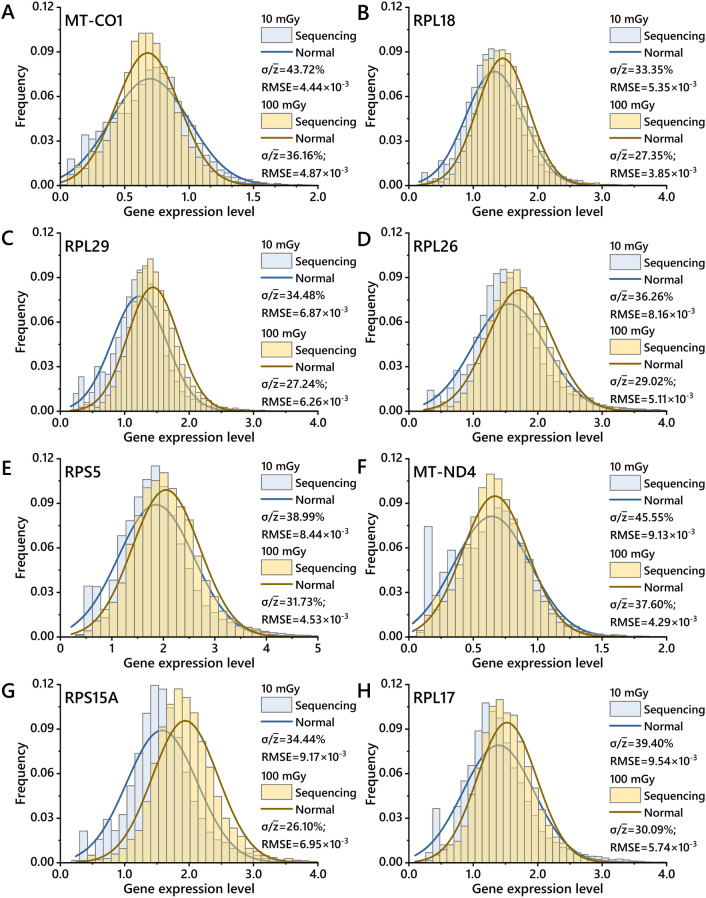
Expression distributions (bar charts) of selected differential genes in BEAS-2B cells irradiated with low doses (10 mGy and 100 mGy) following single-cell sequencing analysis. Normal distributions were obtained by their mean value and standard deviation of expression distributions (solid lines).

Figure 4 displays the expression distribution (bar charts) of the selected differential genes in BEAS-2B cells from the low-dose group (10 mGy and 100 mGy) following single-cell sequencing analysis. It also virtually represents the normal distributions (solid lines) derived from the mean and standard deviations of these expression distributions. The results indicate that some differential genes are sensitive to low-dose radiation, and their expressions undergo numerical statistical fluctuations under low-dose conditions. Supplementary Material S10 lists the average expression levels of several differential genes in the irradiated groups. It can be observed that the expression trends of specific differential genes are inconsistent between the low-dose and high-dose groups. For instance, RPS5, RPS28, and RPS12 were upregulated under low-dose conditions, and their differential expression levels increased with the addition of dose. Conversely, the high-dose group had downregulated expression levels, indicating a negative correlation between radiation dose and differential expression.

### Bioinformatics analysis for multi-filtered genes

**Figure 5:**
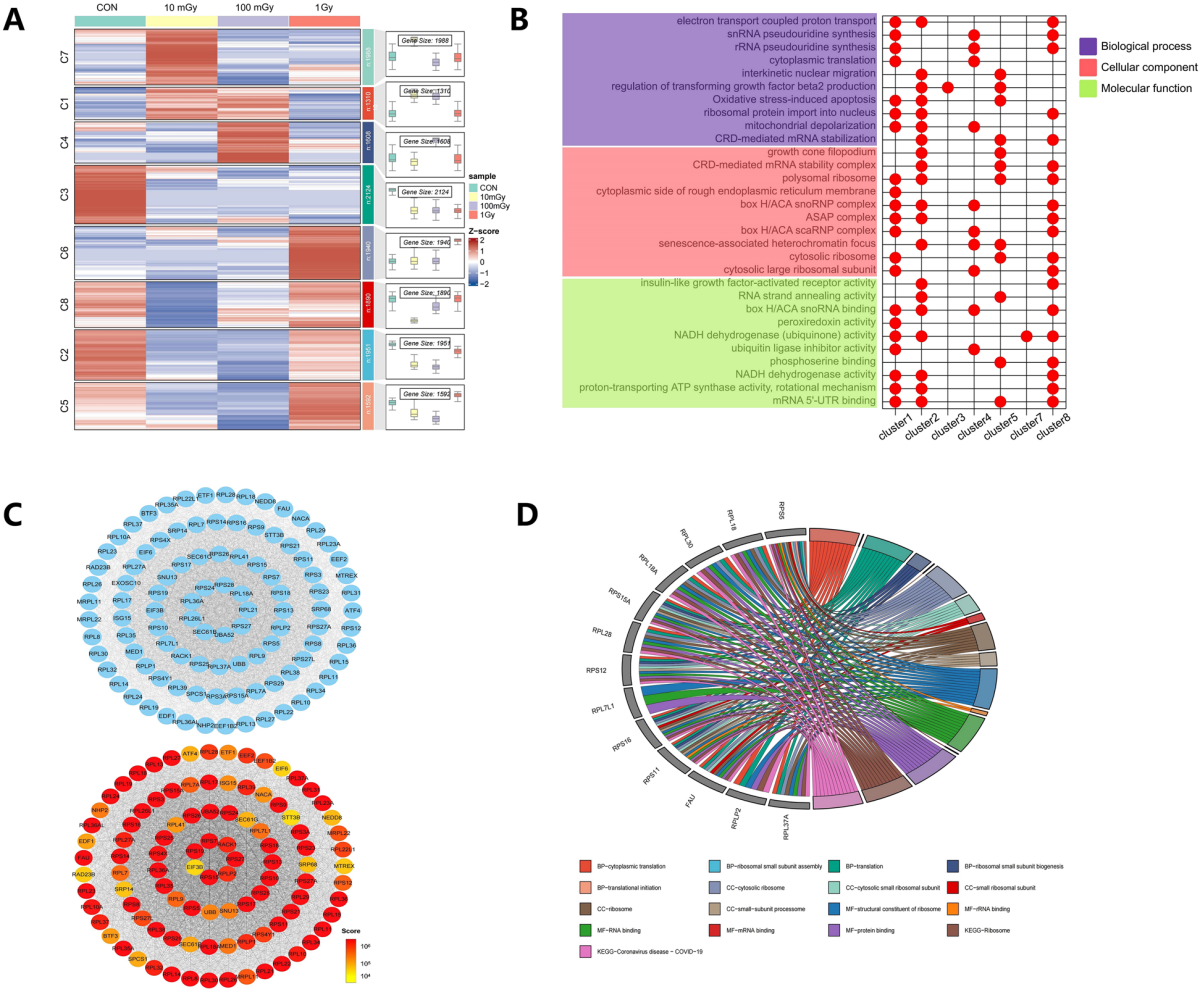
Summary of bioinformatics analysis results. (**A**) Heatmap of dose-dependent transcriptomes among different irradiated groups. (**B**) Top 10 GO analysis results for the combined differential genes from different clusters. (**C**) Interaction network of hub genes obtained through two different methods. (**D**) Chord diagram depicting the top 5 enriched GO pathways and KEGG pathways for the merged 13 genes^42–44^.

We employ the ClusterGVis package and its mfuzz algorithm to segment transcriptome data into distinct clusters based on trends associated with varying irradiation doses. This approach visually illustrates the expression discrepancies among genes in response to different dosage levels. Each gene's alteration trend across different doses is exclusively allocated to a single cluster. 8 gene clusters dependent on irradiation dose were obtained, as shown in Fig. 5A. Specifically, Cluster 3 is the largest cluster with 2124 genes and exhibits a slow decrease in gene expression with increasing doses. However, this trend does not exhibit statistical significance. By comparison, Cluster 1 contains the smallest number of genes (n = 1310), which are more sensitive to lower doses and were upregulated. When the dose reaches 1 Gy, the recovery of gene expression levels is found. According to the criteria of FC > 1.2 and p < 0.05, differential genes were selected from the irradiated groups, and 1222 genes were found to be differentially expressed across all three groups.

The enrichment analysis of differential genes reveals that the Cluster 1 gene set is the most representative among the top ten GO analyses. This cluster primarily includes "Biological Process pathways" such as proton-coupled electron-transfer and snRNA pseudouridine synthesis, "Cell Component pathways" (i.e., polysomal ribosome and cytoplasmic side of rough endoplasmic reticulum membrane), and "Molecular Function pathways", including box H/ACA snoRNA binding, peroxiredoxin activity, etc. (Fig. 5B). Similarly, in the case of the top ten KEGG analyses, the Cluster 1 gene set achieves the highest representativeness. It is identified with various pathways involved in the development of Proteasome, Non-alcoholic fatty liver disease, Prion disease, Thermogenesis, Parkinson's disease, etc. To further explore these differential genes and hub genes within the enriched pathways, the first module and the top 100 hub genes were selected using the MCODE and cytoHubba plugins in Cytoscape 3.9.1 software (as detailed in Supplementary Material S.6). These potentially interacting genes are summarized in Fig. 5C.

The "Statistic merge" results were combined with the differential genes that align with microdosimetric distribution characteristics ("Microdosimetric merge"), as described in Supplementary Material S.6. Multi-filtered genes were primarily from the ribosomal protein family, specifically the PRL and PRS series. The top five GO and KEGG analyses for these genes comprise pathways such as cytoplasmic translation, cytosolic ribosome, rRNA binding, and Ribosome (Fig. 5D). Most of these 13 genes originate from Cluster 1. It is inferred that they may be upregulated and recovered with increasing doses and become increasingly sensitive to low doses, aligning with the trends illustrated in Fig. 5.

## Discussion

The stochastic nature of radiation interactions results in obvious specific energy distributions among cells within a cell population. For this reason, identifying a biological response indicator that can be compared with the specific energy distribution is essential. This interdisciplinary research incorporated microdosimetry and radiation genetics, holding the promise of characterizing the low-dose radiation effects at the molecular level. In addition, it has the potential to screen and develop biomarkers for low-dose radiation responses.

Through simulations and analyzing the specific energy distribution within cell populations for cytoplasm or cell nucleus, the statistical differences in the actual doses received by cells under different radiation parameter conditions can be illustrated. EGS model was employed for cellular-specific energy distributions in PHITS, which can reduce the computational load and be suitable for simulations at the micrometer scale^46^. The mean specific energy, the dispersion of its distribution, and the RMSE derived from a comparison with a normal distribution constitute the three indicators used here, allowing for a partial description of the specific energy distribution. The mesh-type model performs better when reproducing realistic cell morphology than geometry-type cells. The comparison of specific energy distributions from mesh-type and geometric-type cell population models at different initial energies indicates that the model shape and volume impact the dispersion of specific energy distributions. This observation underscores the practical significance of utilizing mesh-type cells, which can closely mimic realistic cells for meticulously constructing cell population models. The type of model changes the dispersions of specific energy distributions but does not affect the average value, offering valuable insights for future work. For instance, employing a geometry-type model would suffice for investigating the specific energy under photon conditions. Moreover, the consideration of specific energy distribution is also crucial. Assuming the presence of a lethal dose for cells, differences in the specific energy distribution within the cell population can lead to biases in evaluating cell survival fractions.

The mean specific energy received by each cell nucleus is not consistent with the macroscopically absorbed and cumulative dose. One of the reasons for this discrepancy may arise from material non-uniformity within cell populations. This observation aligns with findings from Tan et al. in their proton RBE prediction study^47^. Another factor contributing to this difference is the morphology of the cells. Comparing the "Cumulative dose of petri dish with cells" in S3 with the mean specific energy in S5, it can be observed that even after excluding the interference of material, there remains a difference between these two values. Discrepancies between specific energy mean values and macroscopic doses under photon conditions can be found in the results of Oliver et al.^32^.

In several successful and applicable biophysical models^23,25,26,48^, the emphasis is mostly on using absorbed doses that reflect macroscopic averages to represent the degree of irradiation in the cell nucleus. Alternatively, it is assumed that the mean specific energy within domains in the cell nucleus is equal to the macroscopic dose value, while overlooking the fact that cellular materials and morphologies vary in reality. Differences caused by the aforementioned factors would occur in scenarios involving external proton beams^49^. Therefore, it is imperative to engage in precise morphology of realistic cells with high fidelity and their dose assessment.

The MC method was employed to obtain the specific energy distribution within the cell nucleus by simulating the transport behavior of radiation particles within cells. Despite this viable application, it is typically time-consuming, especially for addressing high cumulative macroscopic doses requiring substantial computational resources. Within a range of convolution iterations, convolution offers an efficient method for predicting the specific energy distribution. The premise is that multiple calculations are needed to minimize the error in the distribution before convolution. However, the convoluted distribution becomes distorted with increasing convolutions, particularly for relatively small specific energy ranges. This indicates that even under high-dose irradiation, a large portion of cells may still receive relatively low doses. Moreover, insufficient convolution iterations may lead to deviations in mean specific energy prediction.

The single-cell sequencing data indicate that the expression differences of many differential genes conform to the specific energy distribution features under different irradiation doses. In other words, the response of differential genes reflects the expression fluctuations of specific genes for cells within the same irradiated sample. Based on post-irradiation single-cell sequencing results, we identified a set of differential genes with their expression differential distributions consistent with microdosimetry distribution features. Under the same radiation field conditions, heterogeneity arises in the differential expression of the same gene among different cells, yet this phenomenon is challenging to detect using traditional biological methods, which exclusively use statistical methods for screening differential genes after cell sequencing detection. By comparing gene-level expression distribution with the present microdosimetric distribution, a novel method was developed to identify molecular biomarkers sensitive to low-dose radiation, presenting an efficient approach to selecting differential genes. Furthermore, specific filtered differential genes in the low-dose radiation group exhibited expression levels opposite to those in the high-dose radiation group. This can explain the stimulatory effects in response to low-dose radiation. It has to acknowledge that the biological effects induced by low-dose radiation are the result of the combined effects of multiple factors. We only discussed the distribution features from the perspective of physical doses. However, the indirect effects by chemical reactions also play a crucial role when concerning the biological effects after exposure to photon beams.

A bioinformatics analysis was conducted on the differential genes selected based on microdosimetric distribution characteristics. The dose–response relationship in gene expression lays a solid foundation for the development of biological markers of low-dose radiation, in line with the findings of Yin et al. and Lee et al.^50,51^. To comprehensively evaluate the biological dose effects, sole reliance on individual genes as biological dosimeters may be insufficient. For this reason, a combination of multiple genes and indicators is required for a comprehensive evaluation of the biological dosimetry, as emphasized by Kerns et al.^52^. For this purpose, we selected the top 100 potentially interacting genes from the pool containing 1222 differential genes, providing a valuable reference for future advancement in biodosimetry.

These multi-filtered genes primarily belong to the ribosomal protein family, the NADH-ferricenium reductase family, and the cytochrome c oxidase family. The ribosomal protein family genes are essential ribosomal structural components involved in ribosome assembly and function. In addition to their conventional roles in ribosome function, several ribosomal proteins exhibit extraribosomal functions, activating p53-dependent or p53-independent pathways in response to stress, thus triggering cell cycle arrest and apoptosis^53,54^. Ribosome dysfunctions are implicated in genetic mitochondrial diseases, neurodegenerative conditions, and pathological processes associated with biological aging. Cytochrome c oxidase is a large transmembrane protein complex in bacteria or mitochondria^55,56^. It is sequenced as the fourth complex enzyme within the respiratory electron transport chain, also referred to as Complex IV. Mitochondrial diseases associated with defects in cytochrome c oxidase assembly are considered the most severe among graded mitochondrial disorders. Furthermore, Complex IV-associated gene mutations can cause disorders due to cytochrome c oxidase assembly defects^57,58^.

In sum, incorporating monitoring of ribosomal function or mechanisms of binding between biological macromolecules and gene expression levels in later stages can be a promising biomarker of low-dose radiation sensitivity. This approach may enhance the accuracy and scientific validity of biodosimetry assessments for low-dose radiation.

## Conclusion

Low-dose radiation can induce a wide range of biological effects that are difficult to validate, especially due to the absence of conclusive dose–response relationships. In this study, we constructed a monolayer mesh-type cell population model that faithfully replicates realistic cell culture and irradiation scenarios, along with a simplified geometric cell population model of similar dimensions. We employed Monte Carlo simulations to model and quantify the specific energy distribution within cell nucleus resulting from external photon irradiation under various conditions. These distributions were then compared with normal distributions and those derived from convolution algorithms.

Dosimetry simulations indicated that with increasing doses, the dispersion of specific energy distribution of cell nucleus decreases. Additionally, the specific energy distribution approximates characteristics of a normal distribution when cumulative doses below 100 mGy. Leveraging the microdosimetric distribution features within the low-dose range, we further refined the selection of differential genes meeting statistical requirements following single-cell sequencing.

This work integrates principles and techniques from multiple disciplines, including microdosimetry, radiation genetics, and bioinformatics. Such integration presents a precise method for screening biological markers sensitive to low-dose radiation. It also provides valuable insights for the development of low-dose biological dosimeters in the future.

### Supplementary Information

Supplementary Information.

## Data Availability

Data of single-cell sequencing can be obtained by the GEO repository (No. GSE255800). Correspondence and requests for materials should be addressed to Liang Sun.
