# Supplementary material for: A comparative study on the dose–effect of low-dose radiation based on microdosimetric analysis and single-cell sequencing technology
Source: Sci Rep. 2024 May 21;14:11524. doi: 10.1038/s41598-024-62501-5 (PMC11109114; doi:10.1038/s41598-024-62501-5)

S1. Dimensions of single mesh-type cell models and concentric ellipsoidal geometry-type cell models

| **Single cell model** | | **Geometry-type model** | | **Mesh-type model** | |
| --- | --- | --- | --- | --- | --- |
|  |  | **Cytoplasm** | **Nucleus** | **Cytoplasm** | **Nucleus** |
| **Length of projection (**$\boldsymbol{\mu m}$**)** | **X-axis** | 24.97 | 21.39 | 24.97 | 21.39 |
|  | **Y-axis** | 78.21 | 25.45 | 78.21 | 25.45 |
|  | **Z-axis** | 45.03 | 24.98 | 45.03 | 24.98 |
| **Volume (**$\boldsymbol{\mu m}^{\boldsymbol{3}}$**)** |  | 38905.00 | 7116.54 | 20401.26 | 5147.87 |

S2. Elemental components of nucleus and cytoplasm in the cell population model for MC simulation

| Element | Elemental Mass Composition (%) | |
| --- | --- | --- |
|  | **Nucleus** | **Cytoplasm** |
| Hydrogen | 21.77 | 21.86 |
| Carbon | 12.25 | 13.01 |
| Nitrogen | 2.13 | 1.29 |
| Oxygen | 62.35 | 62.34 |
| Silicon | 0.01 | 0.05 |
| Phosphorus | 0.60 | 0.48 |
| Natrium | 0.04 | - |
| Magnesium | 0.03 | - |
| Sulphur | 0.01 | 0.11 |
| Chlorine | 0.10 | 0.29 |
| Kalium | 0.60 | 0.57 |
| Total | 100.00 | 100.00 |

S3. Particle numbers of simulations for different cumulative dose

| Initial Energy |  | Renormalized dose of petri dish with water | Renormalized dose of petri dish with geometry-type cells | Renormalized dose of petri dish with mesh-type cells |
| --- | --- | --- | --- | --- |
| 100keV |  | 1.00E-09 mGy | 1.06E-09 mGy | 1.20E-09 mGy |
| 200keV |  | 2.35E-09 mGy | 2.45E-09 mGy | 2.76E-09 mGy |
| 500keV |  | 6.39E-09 mGy | 6.83E-09 mGy | 7.58E-09 mGy |
| 1MeV |  | 1.10E-08 mGy | 1.20E-08 mGy | 1.36E-08 mGy |
| 500keV |  | 6.39E-09 mGy | 6.83E-09 mGy | 7.58E-09 mGy |
| 500keV |  | 6.39E-09 mGy | 6.83E-09 mGy | 7.58E-09 mGy |
| 500keV |  | 6.39E-09 mGy | 6.83E-09 mGy | 7.58E-09 mGy |
| 500keV |  | 6.39E-09 mGy | 6.83E-09 mGy | 7.58E-09 mGy |
| 500keV |  | 6.39E-09 mGy | 6.83E-09 mGy | 7.58E-09 mGy |
| Initial Energy | Particle numbers | Cumulative dose of petri dish with water | Cumulative dose of petri dish with geometry-type cells | Cumulative dose of petri dish with mesh-type cells |
| 100keV | 4.98E+09 | 5.00 mGy | 5.34 mGy | 5.99 mGy |
| 200keV | 2.13E+09 | 5.00 mGy | 5.20 mGy | 5.86 mGy |
| 500keV | 7.82E+08 | 5.00 mGy | 5.34 mGy | 5.93 mGy |
| 1MeV | 4.56E+08 | 5.00 mGy | 5.45 mGy | 6.20 mGy |
| 500keV | 3.91E+09 | 25.00 mGy | 26.72 mGy | 29.66 mGy |
| 500keV | 7.82E+09 | 50.00 mGy | 53.45 mGy | 59.32 mGy |
| 500keV | 1.56E+10 | 100.00 mGy | 106.89 mGy | 118.64 mGy |
| 500keV | 3.13E+10 | 200.00 mGy | 213.78 mGy | 237.28 mGy |
| 500keV | 7.82E+10 | 499.99 mGy | 534.45 mGy | 593.21 mGy |

Note that “Renormalized dose of petri dish with water” represents the macroscopic dose of petri dish per radiation particle with the materials of liquid water without cells. “Renormalized dose of petri dish with geometry-type cells” and “Renormalized dose of petri dish with mesh-type cells” represent the macroscopic doses per radiation particle of petri dish filled with geometry-type or mesh-type cells with the cell materials listed in the S2.

S4. Methods of convolution integral

In general, $D_{1}$ represents the absorbed dose of a single event with the distribution of $f_{1}\left( z \right)$. If there exists a specific energy distribution $f_{a}\left( z \right)$ with a dose value of $D_{a}$, it can be considered as the convoluted distribution. when the cumulative dose reaches $D$, $v$ can be determined as follows

$v=D/D_{a}$, (1)

In this case, $D$ values should be set to be divisible by $D_{a}$ so that $v$ should be an integer. As shown in figure 3, $D_{1}$ was 5 mGy, and $D$ were 25 mGy, 50 mGy, 100 mGy, 200 mGy and 500 mGy, whose $v$ were 5, 10, 20, 40, 100, separately, for obtaining convolution distributions.

The multiplicative character of the convolution operation can be utilized to obtain $f_{v}\left( z \right)$ from $f_{a}\left( z \right)$ by a sequence of convolutions that corresponds to the splitting of $v$ into integer powers of 2. For example

$f_{84}\left( z \right)=f_{4}\left( z \right)*f_{16}\left( z \right)*f_{64}\left( z \right)=f_{\left| 2 \right|}(z)*f_{\left| 4 \right|}(z)*f_{\left| 6 \right|}(z)$ (2)

The $\left| v \right|$ represents $2^{v}$ and the $f_{\left| v \right|}(z)$ represents integer powers $2^{v}$ of $f_{a}\left( z \right)$. There are recurrence relations

$f_{\left| 0 \right|}\left( z \right)=f_{a}\left( z \right)$ (3)

S5. Mean specific energies and dispersions of monolayer cell population model

1. Values of mean specific energies and dispersions within the simulated conditions in the Fig.2

|  |  | **Mean specific energy (mGy)** | | **Mean dispersion** | |
| --- | --- | --- | --- | --- | --- |
| **Initial energy** | **Region** | **Mesh-type model** | **Geometry-type model** | **Mesh-type model** | **Geometry-type model** |
| **100keV** | **Cytoplasm** | 5.94±0.01 | 5.88±0.01 | 15.08% | 11.66% |
|  | **Nucleus** | 5.82±0.02 | 5.82±0.04 | 30.98% | 26.02% |
| **200keV** | **Cytoplasm** | 5.89±0.01 | 5.90±0.01 | 17.41% | 13.78% |
|  | **Nucleus** | 5.97±0.05 | 5.86±0.03 | 33.21% | 29.05% |
| **500keV** | **Cytoplasm** | 6.07±0.01 | 5.99±0.01 | 14.26% | 11.99% |
|  | **Nucleus** | 6.09±0.01 | 5.97±0.02 | 26.42% | 23.09% |
| **1MeV** | **Cytoplasm** | 6.29±0.02 | 6.17±0.01 | 13.96% | 13.03% |
|  | **Nucleus** | 6.30±0.05 | 6.13±0.03 | 22.74% | 21.51% |

2. Values of mean specific energies within the simulated conditions in the Fig.3

| **Cumulative dose (mGy)** | **Mean specific energy (mGy)** | **Ratio between mean specific energy and cumulative dose** |
| --- | --- | --- |
| **5** | 5.82 | 1.16 |
| **25** | 27.69 | 1.11 |
| **50** | 59.65 | 1.19 |
| **100** | 119.22 | 1.19 |
| **200** | 238.67 | 1.19 |
| **500** | 596.95 | 1.19 |

S6. Specific energy distributions of cell nucleus and cytoplasm dose in the mesh-type or geometric-type monolayer cell population model for monoenergetic photons with initial energies of 0.1 MeV (A, E), 0.2 MeV (B, F), 0.5 MeV (C, G), and 1 MeV (D, H) at the macroscopic dose level of 5 mGy


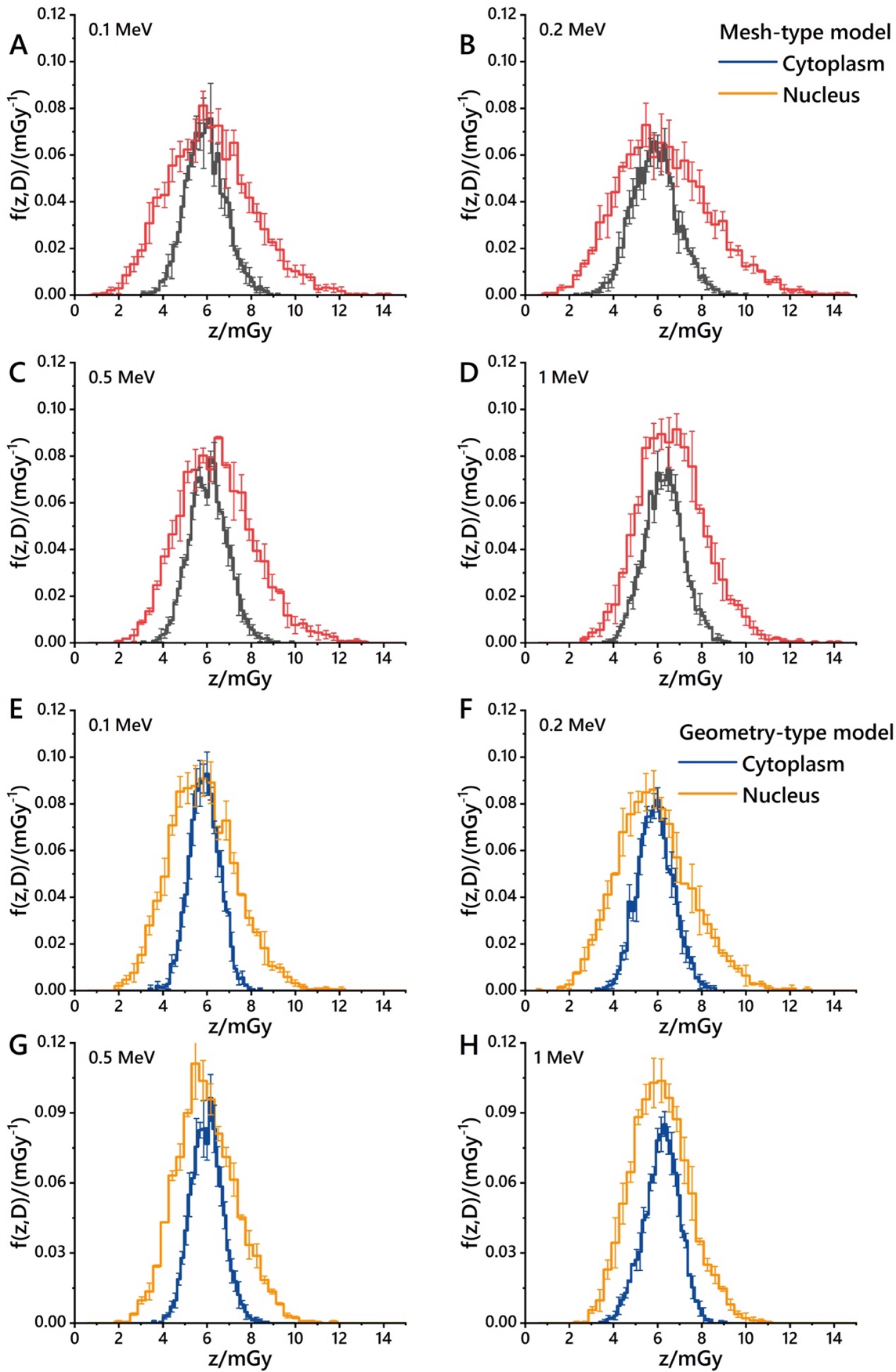


S7. Cell nucleus dose distribution in a monolayer ellipsoidal cell population model with different parameters resulting from external irradiation of Cs-137 monoenergetic photons at various cumulative macroscopic dose levels. (A-B) corresponds to large-sized culture flask, (C-H) to small-sized culture flask, (A-E) represents population models with a high cell number density, and (F-H) represents population models with a low cell number density. For 10 mGy (B, D, and G) and 100 mGy (E, H), the cell nucleus dose distribution includes those obtained through Monte Carlo simulations (red line) and those obtained by convoluting distributions through the same geometric setup conditions at 1 mGy (A, C, F) (brown line), as well as normal distributions fitted to the distributions obtained through Monte Carlo simulations in each case (black line)


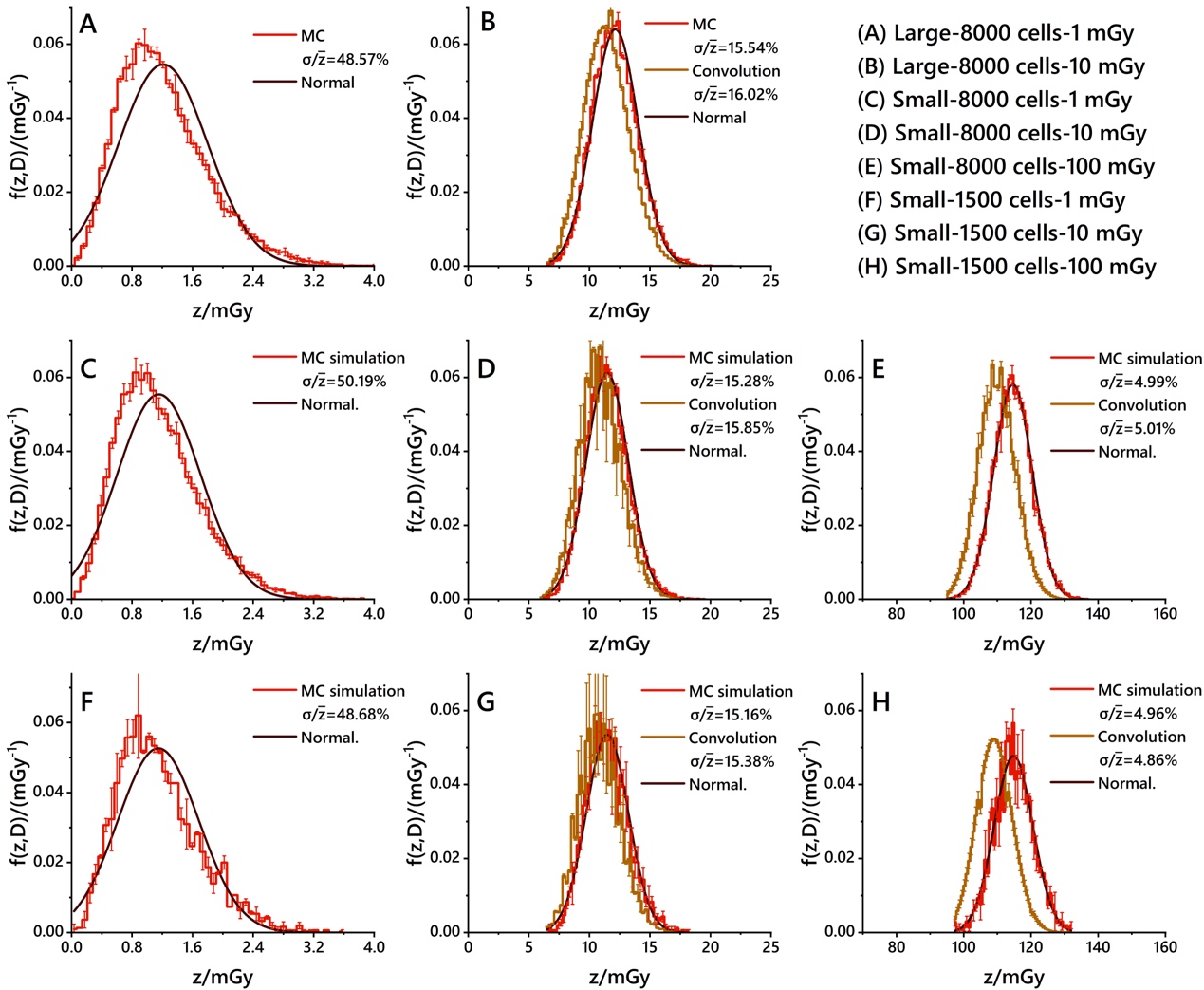


S8. RMSE values obtained by comparing various distributions.

1. RMSE values between two distributions in Figure 2 in the manuscript

|  | Mesh-type model | Geometry-type model |  |
| --- | --- | --- | --- |
| Incident energy | MC vs. normal | MC vs. normal | Distribution figure |
| 100 keV | 3.67E-03 | 3.67E-03 | Figure2 Panel A |
| 200 keV | 3.95E-03 | 3.85E-03 | Figure2 Panel B |
| 500 keV | 4.07E-03 | 4.26E-03 | Figure2 Panel C |
| 1 MeV | 4.23E-03 | 2.49E-03 | Figure2 Panel D |

2. RMSE values between three distributions in Figure 3 in the manuscript

| Cumulative macroscopic dose | MC vs. Normal | MC vs. Convolution | Normal vs. Convolution | Distribution figure |
| --- | --- | --- | --- | --- |
| 5 mGy | 3.20E-03 | - | - | Figure3 Panel A |
| 25 mGy | 1.04E-02 | 2.67E-03 | 9.67E-03 | Figure3 Panel B |
| 50 mGy | 5.97E-03 | 2.34E-03 | 4.96E-03 | Figure3 Panel C |
| 100 mGy | 1.02E-02 | 4.87E-03 | 7.71E-03 | Figure3 Panel D |
| 200 mGy | 1.48E-02 | 8.10E-03 | 1.18E-02 | Figure3 Panel E |
| 500 mGy | 2.64E-02 | 1.44E-02 | 2.04E-02 | Figure3 Panel F |

3. RMSE values between three distributions in S5 in the supplementary material

| Plate size and cell number | Cumulative macroscopic dose | MC vs. Normal | MC vs. Convolution | Distribution figure |
| --- | --- | --- | --- | --- |
| Large-8000 cells | 1 mGy | 6.14E-03 | - | S5 Panel A |
| Large-8000 cells | 10 mGy | 7.88E-03 | 2.06E-03 | S5 Panel B |
| Small-8000 cells | 1 mGy | 6.07E-03 | - | S5 Panel C |
| Small-8000 cells | 10 mGy | 7.45E-03 | 1.98E-03 | S5 Panel D |
| Small-8000 cells | 100 mGy | 1.65E-02 | 1.49E-03 | S5 Panel E |
| Small-1500 cells | 1 mGy | 6.28E-03 | - | S5 Panel F |
| Small-1500 cells | 10 mGy | 7.83E-03 | 3.58E-03 | S5 Panel G |
| Small-1500 cells | 100 mGy | 1.81E-02 | 3.73E-03 | S5 Panel H |

S9. Differential gene names, where “Microdosimetric merge” represents the top 100 differential genes that conform to microdosimetric characteristics, “Statistic merge” represents the top 100 differential genes that meet statistical differences, “Multi-filtered genes” represents the genes that intersect between “Statistic merge” and “Microdosimetric merge”

| No. | Microdosimetric merge | Statistic merge | Multi-filtered genes |
| --- | --- | --- | --- |
| 1 | RPS5 | RPL37A | RPS5 |
| 2 | RPL18 | UBB | RPL18 |
| 3 | RPS23 | NEDD8 | RPS23 |
| 4 | RPL30 | RPL36AL | RPL30 |
| 5 | LGALS1 | RPL30 | RPL18A |
| 6 | RPL18A | EDF1 | RPL39 |
| 7 | RPL39 | RPL28 | RACK1 |
| 8 | RACK1 | RPL36 | RPS15A |
| 9 | RPS15A | RPL18 | RPL28 |
| 10 | RPL28 | RPL18A | RPS18 |
| 11 | RPS18 | EEF2 | RPS21 |
| 12 | RPS21 | RPL31 | RPS12 |
| 13 | RPS12 | SEC61B | RPL7L1 |
| 14 | RPL7L1 | RPL7A | RPS16 |
| 15 | DNMT1 | RPS12 | UBA52 |
| 16 | SF3B2 | RPL7L1 | RPS11 |
| 17 | CTTN | RPS16 | RPS9 |
| 18 | CAPZA1 | RPL8 | FAU |
| 19 | RPS16 | ETF1 | RPL13 |
| 20 | P4HB | RPL35A | RPL27A |
| 21 | UBA52 | MTREX | RPS15 |
| 22 | RPS11 | RPS5 | RPLP2 |
| 23 | RPS9 | RPS4X | RPS28 |
| 24 | FAU | RPL24 | RPL9 |
| 25 | CBX5 | RPL7 | RPL21 |
| 26 | SYNCRIP | RPL23A | RPL37A |
| 27 | RPL13 | RPS27 | RPL7A |
| 28 | RPL27A | RPL11 | RPL17 |
| 29 | POLR2J3 | RPL10 | RPL29 |
| 30 | ZFAS1 | SRP68 | RPS25 |
| 31 | CDC37 | RPL34 | RPL8 |
| 32 | NR3C1 | RPS29 | RPL37 |
| 33 | APLP2 | SEC61G | RPL34 |
| 34 | NFIB | RPS13 | RPS3 |
| 35 | RPS15 | RPL19 | RPS27 |
| 36 | RPLP2 | RPL10A | RPL36 |
| 37 | RPS28 | RPL27 | RPL36A |
| 38 | LRRC59 | RPS19 | RPL11 |
| 39 | RNPS1 | RPS4Y1 | RPS14 |
| 40 | SUMO2 | MRPL11 | RPL26 |
| 41 | RPL9 | RPS17 | RPL7 |
| 42 | RPL21 | RPL17 |  |
| 43 | ACTR3 | EIF3B |  |
| 44 | TMSB10 | RPLP1 |  |
| 45 | S100A13 | RPL35 |  |
| 46 | RPL37A | RPS15A |  |
| 47 | SRSF4 | FAU |  |
| 48 | CD44 | RPS11 |  |
| 49 | USP9X | RPL26 |  |
| 50 | POLR2L | RPLP2 |  |
| 51 | RPL7A | RPL37 |  |
| 52 | RPL17 | RPS3 |  |
| 53 | RPL29 | RPS8 |  |
| 54 | TGOLN2 | RPL23 |  |
| 55 | NONO | EIF6 |  |
| 56 | PRKAR2A | RPS25 |  |
| 57 | HINT1 | RPL29 |  |
| 58 | BRD4 | RPS18 |  |
| 59 | RAD21 | RPS23 |  |
| 60 | HELLS | RPL9 |  |
| 61 | RPS25 | RPL13 |  |
| 62 | RPL8 | RACK1 |  |
| 63 | TLE5 | RPS24 |  |
| 64 | RPL37 | RPL15 |  |
| 65 | HSPA9 | RPL32 |  |
| 66 | NDUFA13 | SPCS1 |  |
| 67 | CYB5R3 | RPL38 |  |
| 68 | PRRC2C | RPS14 |  |
| 69 | G3BP1 | RPS9 |  |
| 70 | HNRNPK | RAD23B |  |
| 71 | RPL34 | SRP14 |  |
| 72 | RPS3 | RPS10 |  |
| 73 | CORO1C | RPL41 |  |
| 74 | RPS27 | EEF1B2 |  |
| 75 | RPL36 | STT3B |  |
| 76 | HSPA4 | RPS3A |  |
| 77 | SSRP1 | NACA |  |
| 78 | CSE1L | SNU13 |  |
| 79 | GNG12 | RPL21 |  |
| 80 | TMSB4X | NHP2 |  |
| 81 | ODC1 | RPS27L |  |
| 82 | SPAG9 | RPL22 |  |
| 83 | ATP5MC1 | ATF4 |  |
| 84 | SSR4 | RPL14 |  |
| 85 | RPL36A | MED1 |  |
| 86 | PEA15 | ISG15 |  |
| 87 | RPL11 | RPL26L1 |  |
| 88 | MYL6 | RPS26 |  |
| 89 | PLEC | MRPL22 |  |
| 90 | RPS14 | RPL22L1 |  |
| 91 | TOMM7 | RPS28 |  |
| 92 | PAWR | RPL36A |  |
| 93 | SON | RPS27A |  |
| 94 | CD63 | RPS7 |  |
| 95 | TRIM44 | RPL27A |  |
| 96 | RPL26 | RPL39 |  |
| 97 | QKI | UBA52 |  |
| 98 | RPL7 | RPS21 |  |
| 99 | UQCRH | BTF3 |  |
| 100 | NF2 | RPS15 |  |

S10. Expression level of selected differential genes in BEAS-2B cells irradiated with different doses (10 mGy, 100 mGy, and 1 Gy) following single-cell sequencing analysis.


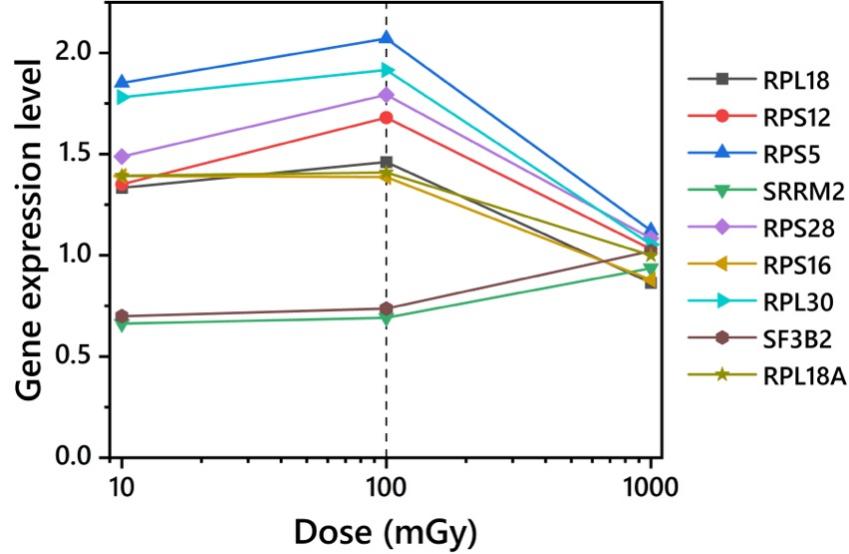

Supplement: Supplementary file 1 — Supplementary Information. [file 41598_2024_62501_MOESM1_ESM.docx]
